# Association between lactate/albumin ratio and all-cause mortality in patients with acute respiratory failure: A retrospective analysis

**DOI:** 10.1371/journal.pone.0255744

**Published:** 2021-08-18

**Authors:** Yan Lu, Haoyang Guo, Xuya Chen, Qiaohong Zhang

**Affiliations:** Clinical Laboratory, DongYang People’s Hospital, Dongyang, Zhejiang, China; Heidelberg University Hospital, GERMANY

## Abstract

Previous studies have shown that lactate/albumin ratio (LAR) can be used as a prognostic biomarker to independently predict the mortality of sepsis and severe heart failure. However, the role of LAR as an independent prognostic factor in all-cause mortality in patients with acute respiratory failure (ARF) remains to be clarified. Therefore, we retrospectively analyzed 2170 patients with ARF in Medical Information Mart for Intensive Care Database III from 2001 to 2012. By drawing the receiver operating characteristic curve, LAR shows a better predictive value in predicting the 30-day mortality of ARF patients (AUC: 0.646), which is higher than that of albumin (AUC: 0.631) or lactate (AUC: 0.616) alone, and even higher than SOFA score(AUC: 0.642). COX regression analysis and Kaplan-Meier curve objectively and intuitively show that high LAR is a risk factor for patients with ARF, which is positively correlated with all-cause mortality. As an easy-to-obtain and objective biomarker, LAR deserves further verification by multi-center prospective studies.

## 1. Introduction

More than 50% of critically ill patients in the intensive care unit (ICU) will suffer from acute respiratory failure (ARF) due to respiratory diseases or pulmonary vascular diseases, accompanied by pulmonary ventilation and/or ventilation dysfunction [1–3]. ARF can cause metabolic disorders and accelerate the deterioration of the underlying condition, which is related to a mortality rate of 35%-46% [4]. Therefore, effective assessment of the prognosis of patients with ARF is of great significance for clinically formulating treatment strategies and improving the survival rate of patients [5].

Due to severe tissue hypoxia in patients with ARF, pyruvic acid cannot be oxidized and is reduced to lactate [6–8]. Therefore, lactate increases in the early stages of ARF. Lactate reflects the imbalance between the supply and demand of oxygen in the organs [9, 10], so the continuous increase in the level of lactate is not only related to hypoxia. ARF causes acute organ hypoxia and vascular endothelial cell damage, which easily induces inflammation [11]. Serum albumin is a negatively regulated acute inflammatory response protein [12]. Previous studies have shown that lactate/albumin ratio (LAR) can be used as a better prognostic biomarker and can independently predict the mortality of critically ill patients, such as sepsis [13, 14] and severe heart failure [15].

The role of LAR as an independent prognostic factor in predicting all-cause mortality in patients with ARF remains to be clarified. Therefore, this study is aim to explore the prognostic value of LAR in predicting the outcome of patients with ARF.

## 2. Materials and methods

### 2.1 Data resource and study population

Medical Information Mart for Intensive Care Database III (MIMIC-III) [16] provides all clinical data of about 50000 unidentified patients at the Beth Israel Deaconess Medical Center (Boston, USA) from 2001 to 2012 for the study, including admission and discharge information, vital signs, laboratory parameters, etc. Author *Lu* gained access to the database and was responsible for data extraction and analysis (certification number: 35953547).

All patients with ARF over 16 years old, who had ICU admission information, were included in the study. Patients with ARF were identified by ICD-9 code and extracted from the MIMIC-III database. The detailed ICD-9 codes were "51851" and "51881". The exclusion criteria included: ① patients without lactate and serum albumin data within 24 hours after admission to ICU; ② Patients who received albumin infusion before entering ICU. In addition, patients with ARF who had repeated admission to ICU only kept the record of the first admission to ICU.

### 2.2 Data extraction

The extraction of MIMIC-III database data was performed in PostgreSQL 10 software. The data extracted for the study include: gender, age, height, weight, first care unit, mechanical ventilation, comorbidities (diabetes, hypertension, chronic obstructive pulmonary disease, heart failure, pneumonia, chronic kidney disease, chronic liver disease, sepsis, and malignancy), vital signs (pH, temperature, oxygen saturation), severity scores (Sequential Organ Failure Assessment (SOFA) [17], Oxford Acute Severity of Illness Score (OASIS) [18], Simplified Acute Physiology Score II (SAPS II) [19]), laboratory parameters (white blood cells, platelet, bicarbonate, creatinine, lactate, albumin). Vital signs and laboratory parameters were extracted within 24 hours after ICU admission. If there were multiple test values, only the first test value could be included.


$$\text{LAR}=\text{lactate}\left(\text{mmol}/\text{L}\right)/\text{serum}\;\text{albumin}\left(\text{g}/\text{dL}\right);\;\text{and}\;\text{body}\;\text{mass}\;\text{index}\left(\text{BMI}\right)=\text{weight}\left(\text{kg}\right)/\text{height}\;\text{squared}\left({\text{m}}^{2}\right).$$

The outcome of this study was 30-day mortality. The survival date starts when the patient enters the ICU.

### 2.3 Statistical analysis

Statistical analysis was performed in Stata software (version 14). Categorical variables are expressed as frequency and percentage. Continuous variables with normal distribution are expressed as mean ± standard deviation, and the differences between groups are analyzed by two-sample t-test [20]. On the contrary, continuous variables with non-normal distribution are expressed as median and interquartile range. The difference was analyzed by Wilcox test. When the *p* value between the two groups was <0.05, it was considered to have a significant difference [21]. Use IBM SPSS Statistics (version 23.0) to draw the receiver operating characteristic (ROC) curve, and the area under the curve (AUC) was used to evaluate the predictive value of LAR for all-cause mortality in patients with ARF [22]. The Youden Index was used to determine the optimal cut-off value. According to the cut-off value, LAR was divided into low LAR group and high LAR group.

The survival difference between the high LAR group and the low LAR group was shown by Kaplan-Meier curve [23]. Three cox regression models were used to prove the independent correlation between LAR and all-cause mortality. Model I was unadjusted; Model II was adjusted by gender, age, BMI, and first care unit; based on model II, Model III added mechanical ventilation, comorbidities (diabetes, hypertension, chronic obstructive pulmonary disease, heart failure, pneumonia, chronic kidney disease, chronic liver disease, sepsis, and malignancy), vital signs (temperature, pH, oxygen saturation), severity scores (SOFA, OASIS, SAPS II), laboratory parameters (white blood cells, bicarbonate, creatinine, and platelets) as covariates.

## 3. Results

Initially, 7490 patients with ARF were screened from the MIMIC-III database. According to the inclusion and exclusion criteria, 2170 patients were finally enrolled in the study cohort, including 1351 survivors and 819 non-survivors. As shown in Table 1, there was a significant difference in LAR levels between the survivor group and the non-survivor group [0.577 (0.387–1) vs. 0.857 (0.519–1.636), *p* <0.001].

**Table 1:** Patient characteristics of the survivor group and the non-survivor group.

Variables	Survivors (n = 1351)	Non-survivors (n = 819)	*P*-value
Age, years	61.89 (49.27–74.47)	70.22 (57.70–81.21)	<0.001
Male, n (%)	721 (53.37)	470 (57.39)	0.068
BMI, kg.m^-2^	27.46 (23.48–32.53)	26.25 (22.46–30.95)	<0.001
First care unit, n (%)			0.213
Coronary Care Unit	152 (11.25)	102 (12.45)	
Cardiac Surgery Intensive Care Unit	61 (4.52)	29 (3.54)	
Medicine Intensive Care Unit	856 (63.36)	539 (64.81)	
Surgical Intensive Care Unit	183 (13.55)	87 (10.62)	
Trauma Surgery Intensive Care Unit	99 (7.33)	62 (7.57)	
Mechanical Ventilation, n (%)	1238 (91.64)	725 (88.52)	0.017
Comorbidities, n (%)			
Diabetes	420 (31.09)	214 (26.13)	0.014
Hypertension	495 (36.64)	266 (32.48)	0.049
chronic obstructive pulmonary disease	66 (4.89)	33 (4.03)	0.354
Heart failure	140 (10.36)	71 (8.67)	0.197
Pneumonia	586 (43.38)	300 (36.63)	0.002
Chronic kidney disease	235 (17.39)	167 (20.39)	0.082
Chronic liver disease	79 (5.85)	45 (5.49)	0.731
Malignancy	117 (8.66)	193 (23.57)	<0.001
Sepsis	979 (72.46)	618 (75.46)	0.125
Vital signs			
pH	7.34 (7.26–7.41)	7.32 (7.22–7.4)	<0.001
SPO_2_, %	90 (85–98)	88 (85–98)	0.1142
Temperature, °C	36.89 (36.28–37.5)	36.55 (35.83–37.44)	<0.001
Severity scores			
SOFA	6 (4–9)	8 (5–11)	<0.001
OASIS	38.49±7.99	42.86±8.89	<0.001
SAPSII	42 (33–52)	54 (45–66)	<0.001
white blood cell, 10 ^9^/L	11.9 (7.9–17.1)	13.1 (8.5–19.5)	<0.001
Platelet, 10 ^9^/L	206 (142–282)	191 (110–285)	<0.001
Bicarbonate, mmol/L	22 (19–26)	20 (17–24)	<0.001
Creatinine, mg/dL	1.1 (0.8–2)	1.4 (0.9–2.3)	<0.001
Albumin, g/dL	3 (2.6–3.4)	2.6 (2.2–3.1)	<0.001
Lactate, mmol/L	1.7 (1.2–2.6)	2.2 (1.4–4.1)	<0.001
LAR	0.577 (0.387–1)	0.857 (0.519–1.636)	<0.001

SPO_2_: oxygen saturation; SOFA: Sequential Organ Failure Assessment; OASIS: Oxford Acute Severity of Illness Score; SAPS II: Simplified Acute Physiology Score II.

**Fig 1:**
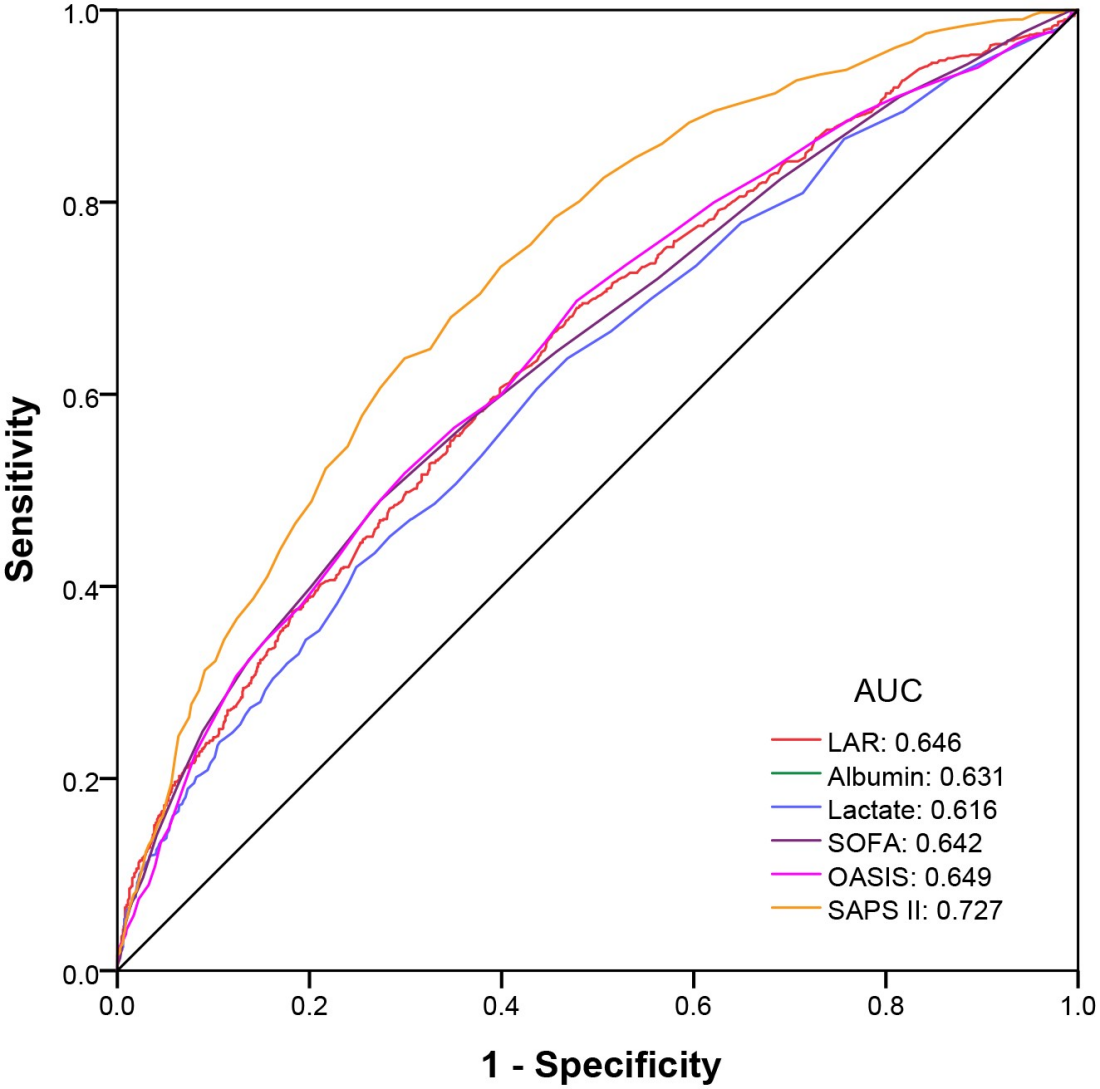
ROC curve of 30-day mortality in patients with ARF.

The ROC curve was analyzed for the predictive value of LAR in the 30-day mortality of ARF patients (Fig 1). The predictive value of LAR [AUC: 0.646, 95% confidence interval (CI) 0.622–0.670] was better than that of lactate (AUC: 0.616, 95% CI 0.592–0.641) or albumin (AUC: 0.631, 95% CI 0. 0.607–0.655) alone, and even higher than that of SOFA (AUC: 0.642, 95% CI 0.618–0.666). According to the Youden Index, the optimal cut-off point of LAR was 0.597 with a sensitivity of 0.690, and a specificity of 0.522. Compared with SOFA and SAPS II, LAR has better sensitivity in predicting the 30-day mortality of ARF (sensitivity: 0.690 vs. 0.490, 0.637) (Table 2).

**Table 2:** Diagnostic performance of LAR, albumin, lactate, SOFA, OASIS, and SAPS II in the 30-day mortality of ARF patients.

	AUC	95% CI	Sensitivity	Specificity
LAR	0.646	0.622–0.670	0.690	0.522
Albumin	0.631	0.607–0.655	0.696	0.503
Lactate	0.616	0.592–0.641	0.420	0.751
SOFA	0.642	0.618–0.666	0.490	0.726
OASIS	0.649	0.625–0.673	0.697	0.522
SAPS II	0.727	0.706–0.749	0.637	0.701

According to the optimal cut-off point of LAR, the study population was divided into the high LAR group (LAR ≥ 0.597) and low LAR group (LAR < 0.597). There were 1211 people in the high LAR group and 959 people in the low LAR group. There were significant differences in age, comorbidities, severity scores, and laboratory parameters between the two groups (Table 3).

**Table 3:** Patient characteristics of the study patients according to LAR levels.

Variables	Low LAR group	High LAR group	*P*-value
	(n = 959)	(n = 1211)	
LAR	0.406 (0.313–0.5)	1.087 (0.792–1.8)	<0.001
Age, years	64.61 (52.60–77.24)	65.71 (51.75–78.31)	0.409
Male, n (%)	504 (52.55)	687 (56.73)	0.052
BMI, kg.m^-2^	27.02 (23.05–31.99)	26.98 (23.11–31.64)	0.522
First care unit, n (%)			0.087
Coronary Care Unit	120 (12.51)	134 (11.07)	
Cardiac Surgery Intensive Care Unit	33 (3.44)	57 (4.71)	
Medicine Intensive Care Unit	622 (64.86)	773 (63.83)	
Surgical Intensive Care Unit	126 (13.14)	144 (11.89)	
Trauma Surgery Intensive Care Unit	58 (6.05)	103 (8.51)	
Mechanical Ventilation, n (%)	853 (88.95)	1110 (91.66)	0.033
Comorbidities, n (%)			
Diabetes	319 (33.26)	315 (26.01)	<0.001
Hypertension	358 (37.33)	403 (33.28)	0.049
chronic obstructive pulmonary disease	68 (7.09)	31 (2.56)	<0.001
Heart failure	112 (11.68)	99 (8.18)	0.006
Pneumonia	440 (45.88)	446 (36.83)	<0.001
Chronic kidney disease	204 (21.27)	198 (16.35)	0.003
Chronic liver disease	41 (4.28)	83 (6.85)	0.010
Malignancy	115 (11.99)	195 (16.10)	0.007
Sepsis	661 (68.93)	936 (77.29)	<0.001
Vital signs			
pH	7.35 (7.28–7.42)	7.32 (7.23–7.39)	<0.001
SPO2, %	88 (85–98)	90 (85–98)	0.019
Temperature, °C	36.89 (36.28–37.44)	36.72 (36–37.5)	0.018
Severity scores			
SOFA	5 (4–8)	8 (5–11)	<0.001
OASIS	38.04±7.94	41.80±8.75	<0.001
SAPSII	41 (33–51)	51 (41–62)	<0.001
white blood cell, 10 ^9^/L	11.4 (8–16.6)	13.2 (8.1–19.5)	<0.001
Platelet, 10 ^9^/L	217 (157–291)	183 (113–274)	<0.001
Bicarbonate, mmol/L	24 (20–28)	20 (16–24)	<0.001
Creatinine, mg/dL	1.1 (0.7–2)	1.3 (0.9–2.2)	<0.001
Albumin, g/dL	3.1 (2.7–3.5)	2.6 (2.2–3.1)	<0.001
Lactate, mmol/L	1.2 (1–1.5)	2.9 (2.1–4.7)	<0.001
30-day mortality	254 (26.49)	565 (46.66)	<0.001

**Fig 2:**
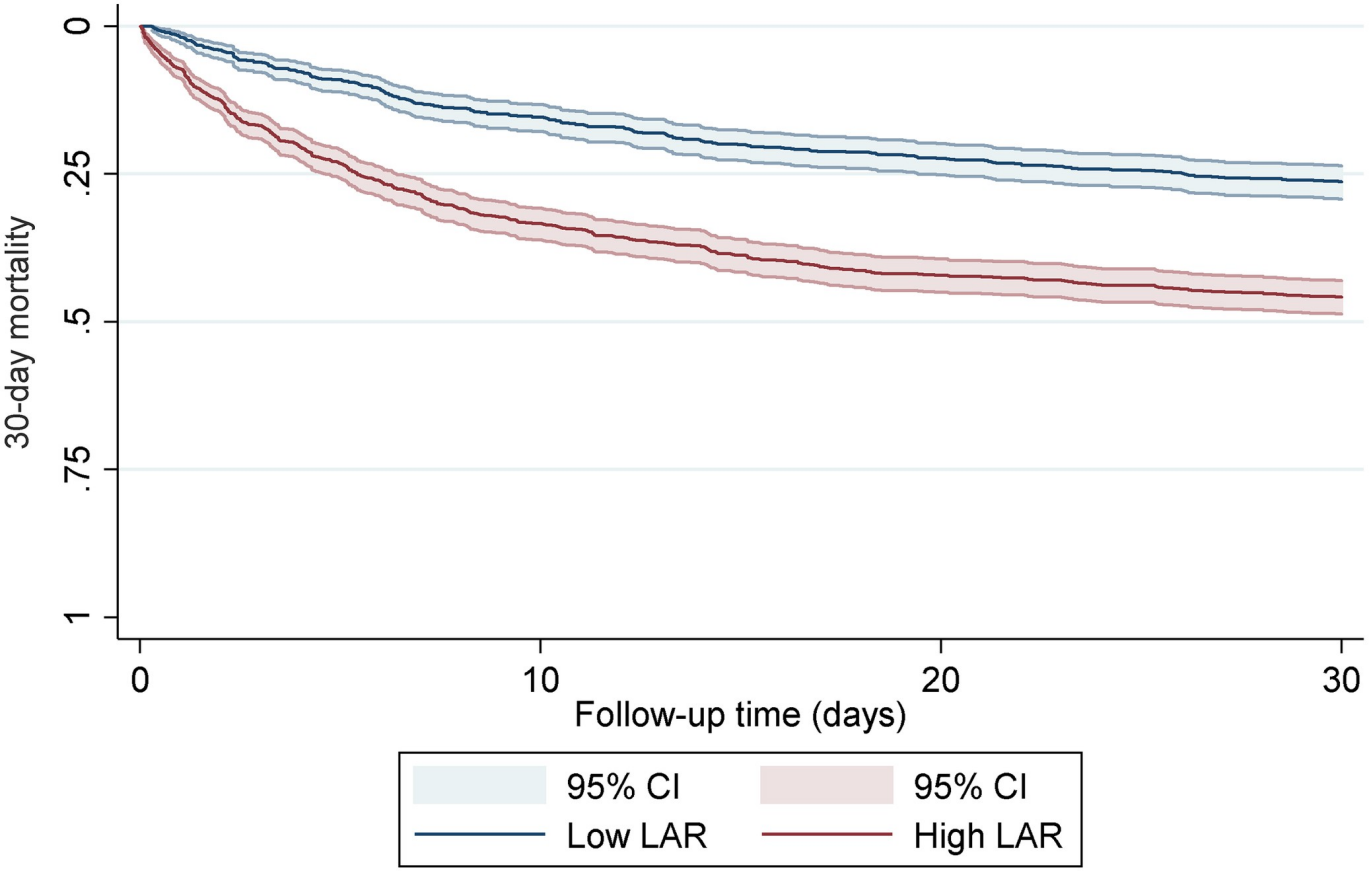
Kaplan-Meier curves of 30-day all-cause mortality for patients with ARF.

As shown in Fig 2, the Kaplan-Meier curve shows that the 30-day all-cause mortality in the high LAR group was significantly higher than that in the low LAR group. In the COX Regression Model I, compared with the low LAR group, the unadjusted hazard ratio (95% CI) of the high LAR group was 2.12 (1.82–2.45). After Model II adjusted gender, age, BMI, and first care unit, the higher risk in the high LAR group remained unchanged, and the hazard ratio (95% CI) was 2.06 (1.77–2.39). Model III was adjusted by adding 23 covariates, and the rising trend of the risk of the high LAR group was still significant, and the hazard ratio (95% CI) was 1.56 (1.33–1.83) (Table 4).

**Table 4:** Hazard ratio (95% confidence interval) of 30-day all-cause mortality according to groups of LAR levels.

	30-day mortality		
	Hazard ratio	95% Confidence Interval	*P*-value
Model I	2.12	1.82–2.45	<0.001
Model II	2.06	1.77–2.39	<0.001
Model III	1.56	1.33–1.83	<0.001

Model I: Non-adjusted.

Model II: Adjusted by gender, age, BMI, and first care unit.

Model III: Adjusted by gender, age, BMI, first care unit, diabetes, chronic obstructive pulmonary disease, heart failure, pneumonia, hypertension, chronic kidney disease, chronic liver disease, malignancy, sepsis, mechanical ventilation, temperature, pH, SPO_2_, SOFA, OASIS, SAPS II, white blood cells, bicarbonate, creatinine and platelet.

## 4. Discussion

This study has some interesting findings. First, after adjusting for confounding factors using multivariate COX regression analysis, LAR is still an independent risk factor for 30-day mortality in patients with ARF. Second, LAR has shown similar predictive value to the commonly used scoring systems for critically ill patients such as SOFA and OASIS. Finally, patients with high LAR have a significantly higher risk of 30-day mortality than patients with low LAR.

As far as we know, this is the first study focusing on the relationship between LAR and the prognosis of patients with ARF. Currently, severity scores are widely used clinically to evaluate the prognosis of patients [24–26]. However, compared with the severity score composed of multiple clinical factors, LAR has the same superior performance in assessing the prognosis of patients with ARF, similar to SOFA and OASIS. In addition, both lactate and albumin are easily obtained laboratory results and are objective values. Therefore, LAR has fewer restrictions in clinical application and is easier to be accepted clinically.

Patients with ARF have hypoxemia and (or) hypercapnia due to respiratory dysfunction, resulting in blockage of aerobic metabolic pathways. Lactate is a product of anaerobic respiration, which is produced in large amounts under hypoxia, and is positively correlated with mortality [27, 28]. Most patients with ARF die from the progressively worsening pulmonary gas exchange obstruction and diffuse pulmonary inflammation [29]. Albumin promotes the formation of anti-inflammatory substances such as lipoxins, resolvins and protectins, which can promote wound healing and inhibit disease progression [30]. This may explain the poor prognosis caused by albumin reduction. Many studies have shown that albumin has potential as a prognostic marker of ARF [31–33]. Lactate and albumin are regulated by different mechanisms, so LAR can reduce the influence of a single factor on the regulation mechanism [34]. Our study also proved that the predictive value of LAR can be better than lactate or albumin alone.

LAR is not a novel prognostic marker proposed for the first time. In 2015, *Biao Wang* et al. proposed that high LAR would accelerate multiple organ failure and increase mortality in patients with severe sepsis [35]. Since then, LAR has been widely analyzed as a risk stratification tool for critically ill patients [34]. However, the condition of critically ill patients in the ICU is complex and the main causes are quite heterogeneous. Therefore, we think that it is not a trustworthy practice to include all critically ill patients in the ICU with a unified standard. Acute respiratory failure is one of the main causes of short-term mortality in critically ill patients, but most of them have multiple comorbidities. Therefore, whether LAR is an independent prognostic factor for the prognosis of ARF is still controversial. This study demonstrated the potential of high LAR as an independent risk factor for ARF through multiple models.

We cannot deny that our results have limitations. First, this is a single-center retrospective study. It is difficult to obtain data using blind methods and to follow the principle of randomness. As a result, there may be potential biases caused by other interfering factors. Second, the data was collected from 2001 to 2012. We cannot ignore the improvement of medical technology such as the continuous improvement of ICU management level and the continuous optimization of patient treatment plans. It is unknown whether this will affect the results. Therefore, we need a multi-center prospective study to verify our results.

## 5. Conclusion

High lactate/albumin ratio is an independent risk factor for all-cause mortality in patients with ARF. As an easy-to-obtain and objective biomarker, LAR deserves further verification by multi-center prospective studies.
